# The Interactions between Smoking and Sleep

**DOI:** 10.3390/biomedicines12081765

**Published:** 2024-08-05

**Authors:** Ioanna Grigoriou, Serafeim-Chrysovalantis Kotoulas, Konstantinos Porpodis, Dionysios Spyratos, Ioanna Papagiouvanni, Alexandros Tsantos, Anastasia Michailidou, Constantinos Mourelatos, Christina Mouratidou, Ioannis Alevroudis, Alexandra Marneri, Athanasia Pataka

**Affiliations:** 1 Respiratory Failure Clinic and Sleep Laboratory, General Hospital of Thessaloniki “G. Papanikolaou”, Aristotle’s University of Thessaloniki, 54642 Thessaloniki, Greece; ioagrig@hotmail.gr (I.G.); patakath@yahoo.gr (A.P.); 2 Adult ICU, General Hospital of Thessaloniki “Ippokrateio”, 54642 Thessaloniki, Greece; chris1mourat@gmail.com (C.M.); giannis.alevroudis@gmail.com (I.A.); alexia.mrn@gmail.com (A.M.); 3 Pulmonary Department, General Hospital of Thessaloniki “G. Papanikolaou”, Aristotle’s University of Thessaloniki, 54642 Thessaloniki, Greece; kporpodis@yahoo.gr (K.P.); diospyrato@yahoo.gr (D.S.); 4 4th Internal Medicine Department, General Hospital of Thessaloniki “Ippokrateio”, Aristotle’s University of Thessaloniki, 54642 Thessaloniki, Greece; ioanna.d.pap@gmail.com; 5 Pulmonary Department, General Hospital of Thessaloniki “Ippokrateio”, 54642 Thessaloniki, Greece; alextsantos@yahoo.com; 6 2nd Propaedeutic Internal Medicine Department, General Hospital of Thessaloniki “Ippokrateio”, Aristotle’s University of Thessaloniki, 54642 Thessaloniki, Greece; manastasia96@gmail.com; 7 Genetics Laboratory, Aristotle’s University of Thessaloniki, 54124 Thessaloniki, Greece; mourelatos@yahoo.com

**Keywords:** smoking, tobacco, sleep, sleep disorders

## Abstract

Smoking a cigarette before bed or first thing in the morning is a common habit. In this review, the relationship between smoking and sleep is investigated based on the existing literature. Out of 6504 unique items that were identified via a PubMed search related to smoking and sleep, 151 were included in this review. Tobacco smoking disrupts sleep architecture by reducing slow wave and rapid eye movement (REM) sleep and undermining sleep quality. Furthermore, smoking affects sleep-related co-morbidities, such as obstructive sleep apnea–hypopnea syndrome (OSAHS), insomnia, parasomnias, arousals, bruxism, and restless legs, as well as non-sleep-related conditions such as cardiovascular, metabolic, respiratory, neurologic, psychiatric, inflammatory, gynecologic and pediatric issues, while poor sleep quality also seems to worsen the chances of successful smoking cessation. In conclusion, the existing literature suggests that there is a wicked relation between smoking and sleep.

## 1. Introduction

The majority of smokers start their day with smoking one or more cigarettes. Smoking after waking up in the morning is a strong marker of nicotine addiction. A last cigarette before going to bed at night is also another common habit among smokers. Smoking before and after sleep is associated with the nadir in nicotine levels at night due to smoking abstinence during sleep time. However, smoking and sleep are not irrelevant to each other. There are many studies which have demonstrated the relationship between smoking and sleep quality [1,2,3,4]. Poor sleep quality seems to be related to increased craving for tobacco smoke and reduced chances of successful smoking cessation [2], while smoking itself alters sleep architecture [3], and has detrimental effects on sleep quality [4]. Furthermore, smoking seems to worsen many aspects of obstructive sleep apnea–hypopnea syndrome (OSAHS) [5], while it is also related to other sleep disorders like insomnia [6], parasomnias [7], arousals [8], bruxism [9] and restless legs [10]. In addition, poor sleep or OSAHS along with smoking have a multiplicative action in several other medical conditions, such as obesity and metabolic syndrome [11,12], cardiovascular, respiratory and neuropsychiatric diseases [13,14,15,16], and complications in pregnancy or in infant growth [17,18]. The aim of this review is to shed light on all these deleterious interactions between tobacco smoking and sleep. The rationale for conducting this review was to consolidate the existing knowledge about the relationship between smoking and sleep and to present it in separate thematic sectors regarding the relationship of smoking with sleep architecture and quality, OSAHS and other sleep disorders or medical conditions and the effect of poor sleep on smoking cessation.

## 2. Methods

**Figure 1:**
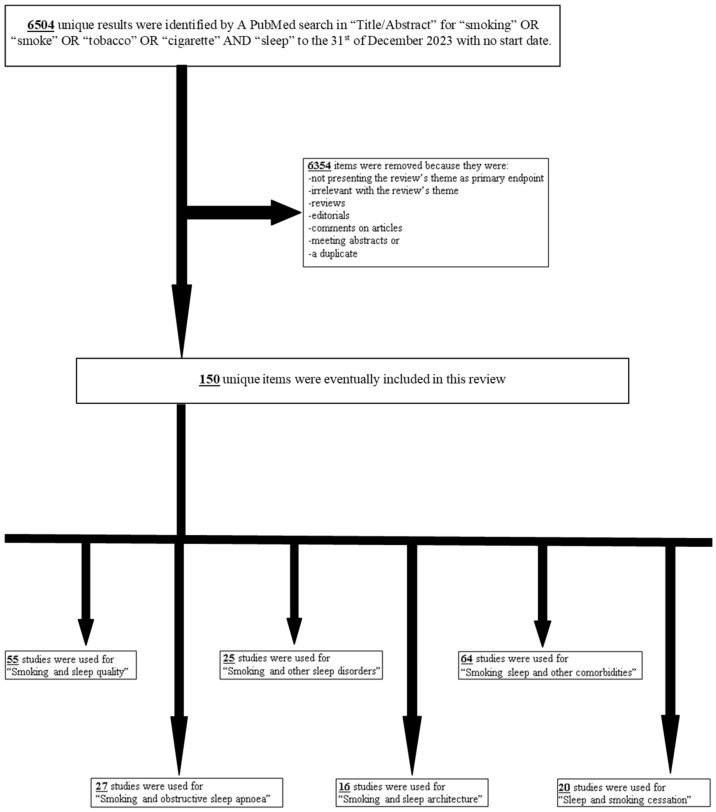
Flow diagram of study selection progress. Some of the studies included, were used in the answers of more than one question of the present review.

For the purpose of the present review, we performed a PubMed search in “Title/Abstract” for “smoking” OR “smoke” OR “tobacco” OR “cigarette” AND “sleep” to the 31st of December 2023 with no start date. This search identified 6504 unique results. Items were removed if they were reviews, editorials, comments on articles, meeting abstracts, a duplicate, irrelevant to the research theme or had not among the primary endpoints the relationship between smoking and sleep. As a result, out of 6504 unique items, 6353 were removed and 151 were included in this review. Articles were categorized based on the thematic sectors of this review. Some of the studies included were used in more than one thematic sector. For ‘smoking and sleep quality’, 55 studies were used. For the ‘smoking and obstructive sleep apnea’ sector, 27 studies were used. For ‘smoking and other sleep disorders’, 25 studies were used. For ‘smoking and sleep architecture’, 16 studies were used. For ‘smoking, sleep and other comorbidities’, 64 studies were used. Finally, 21 studies were used for the ‘sleep and smoking cessation’ sector. The consort diagram is shown as Figure 1. For any studies written by one or more co-authors of the present review, an independent author, who had not participate in those studies, reviewed them for suitability of inclusion.

## 3. Discussion

### 3.1. Smoking and Sleep Quality

The stimulant effect of nicotine was reported more than 40 years ago [19]. Back in the 1980s and 1990s, several studies had correlated smoking with various aspects of poor sleep quality, such as difficulty in initiating sleep, staying asleep and waking up, nightmares, snoring, excessive daytime sleepiness and minor accidents [20,21,22]. Sleep duration was inversely correlated with smoking even in athletes [23], whilst the effects of smoking on sleep were partly attributed to the fact that smokers consume greater quantities of caffeine and alcohol as well [24]. However, in a longitudinal, population-based cohort study with 7960 participants, it was found that adolescents that had no sleep problems previously reported sleep disorders at follow-up, showing a dose–response relationship with smoking [25].

Numerous studies have been published on this topic; in many of them, the main tool used to measure sleep disturbance was the Pittsburgh Sleep Quality Index (PSQI). In these studies, PSQI scores were persistently higher in smokers compared to non-smokers [26,27,28]. The same outcome also concluded a 6-week double-blind randomized controlled trial which investigated the possible augmented effects of naltrexone to nicotine patch in smoking cessation [29]. Furthermore, in two large cross-sectional surveys from Korea, which included cumulatively almost 400,000 participants, the same relationship was also found for both men and women separately [1], while in the latter, the PSQI in smokers remained significantly higher after controlling for psychological factors as well [30]. Other study, which also used the PSQI, described a quantitative relation of sleep disturbance and smoking with significantly longer sleep latency in heavy smokers compared to non-smokers [31,32]. In a 12-week randomized controlled trial about the relationships between exercise, sleep, and smoking, higher PSQI scores were correlated with increased withdrawal, craving and total smoking urges assessed by the Minnesota Nicotine and Withdrawal Scale (MNWS) [4].

Apart from the PSQI, there are also other sleep parameters that present deterioration with smoking. A study which examined the difficulty falling asleep (chronic or recent), difficulty staying asleep, and weekday and weekend sleep duration in relation with past 30-day smoking, found significant reciprocal, prospective relationships between smoking and sleep problems which were more evident to the black race compared to Caucasians [33]. Longer sleep latency, shorter sleep time and difficulty in maintaining sleep seem to present consistently more frequently in smokers compared to non-smokers in numerous studies [6,34,35,36]. However, there was conflicting evidence on the difficulty in initiating sleep and awakenings earlier than desired [35,36]. In any case, many studies have demonstrated that sleep problems associated with smoking, come in a dose–response manner [37]. For example, e-cigarette users have lower odds to present inadequate sleep duration compared to cigarette users, while dual users present the highest odds [38], and the same goes for heavy smokers compared to regular smokers [39], or those who consume tobacco with higher nicotine concentrations [40]. Furthermore, there is increasing evidence that those who are exposed to second-hand smoke are also more prone to develop sleep disturbances, such as short, insufficient or poor-quality sleep, than those who are not [41,42,43,44]. Another factor that possibly intervenes in the relationship between smoking and sleep disturbances is stress [45], something that was even more evident during the COVID-19 pandemic [46]. Finally, chronotype and shiftwork seem to also play some role in this relationship, with those who belong to late chronotypes and night shiftworkers being more vulnerable in the exacerbation of poor sleep quality due to smoking [17,47,48].

On the other hand, there are also studies that have not identified significant relationships between cigarette smoking and sleep quality, apart from an indirect confounding action along with other socioeconomic factors [49], or merely an insignificant effect in sleep hygiene [50]. However, all the aforementioned studies used mainly questionnaires, whilst the detrimental effect of smoking on sleep quality has also been demonstrated with biochemical and genetic examinations. More particularly, urine cotinine and 1-hydroxypyrene have been found increased in smokers with long sleep latency, short sleep time and poor sleep quality overall, compared to never or passive smokers [51,52]. Moreover, a genetic study found negative genetic correlations between smoking initiation and sleep duration and smoking cessation and chronotype, while positive genetic correlations between smoking initiation and cigarettes per day with insomnia [53]. Yet another aspect in the relationship between smoking and poor sleep quality is their common effect on other unhealthy habits. In fact, many studies have shown this effect in bad nutrition, caffeine intake, alcohol consumption, illicit drug usage and lack of physical activity [54,55,56,57,58,59,60].

Since the majority of the studies that were included, so far, in this review are cross-sectional, a temporal relationship between smoking and poor sleep quality cannot be established. Thus, the boundaries are vague as far as the causality between these two. Two large longitudinal studies that investigated the relation between sleep problems in adolescence and subsequent smoking trajectories resulted in conflicting evidence, since the one found a significant relationship [61], whilst the other did not [62]. Another similar study linked poor sleep adequacy in adolescence with subsequent increased smoking behavior through delayed reward discounting mechanism due to adverse childhood experiences [63]. In any case, it seems that sleep deprivation or a transition from adequate to inadequate sleep causes increased tobacco cigarette consumption, probably due to the expectance that nicotine might reduce subjective sleepiness [64,65]. This might also explain why night shiftworkers smoke more [66], or why poor sleep quality is associated with lower quit attempt efficacy [67]. Due to all that, it was suggested that smoking cessation programs should target smokers with poor sleep and promote interventions in this direction [68,69]. Table 1 shows the studies that investigated the relationship between smoking and sleep quality.

### 3.2. Smoking and Obstructive Sleep Apnea

Various pathophysiological mechanisms have been proposed to explain the relationship between smoking and OSAHS. Increased thickness and edema, along with positive staining for calcitonin gene-related peptide (CGRP)—a neuroinflammatory marker for peripheral nerves—have been found in the uvular mucosa lamina propria of smokers. This suggests that smoking might worsen OSAHS through exacerbation of upper airway collapse at the level of the uvula via CGRP neurogenic inflammation leading to increased apnea–hypopnea and oxygen desaturation indices (AHI and ODI) [70]. Another mechanism is that of increased nasal mucociliary clearance time in smokers, with a dose-dependent manner [71]. Furthermore, increased total nasal resistance in a supine position also seems to play a role in smokers with history of habitual snoring [72].

Nevertheless, there is conflicting evidence on the relationship between smoking and OSAHS prevalence. A study which used STOP-Bang, found that former and current male smokers had moderately and severely increased risk for OSA, respectively [73]. In another study, OSAHS was more prevalent in smokers compared to non-smokers [74], while in two more studies current, but not former smokers, presented a higher odds ratio in a dose-dependent way for sleep-disordered breathing or OSAHS, adjusted for confounders such as age, sex, body mass index (BMI) and alcohol [75,76]. However, in three other studies, smoking was not related to the prevalence of OSAHS, when adjusted for the same covariates [13,77,78], apart from in younger females in one study [77], while OSAHS severity was related to smoking in patients with BMI < 30 in another [78].

As far as the relationship between smoking and OSAHS severity, things are rather clearer. Current smokers seem to present OSAHS earlier than their non-smoking counterparts [79]. Smokers also seem to present more severe OSAHS, at least in the majority of the studies [5,7,79,80,81,82,83], compared to non-smokers. Numerous OSA indices were deteriorated in smokers including ODI, mean and minimum SaO_2_, total sleep time and sleep time ratio with SaO_2_ below 90%, mean apnea duration, nocturnal hypoxia index and COHb levels [5,7,79,80,81,82,83,84,85,86,87,88]. However, the relationship of AHI with smoking is not clear. Many studies concluded that AHI is deteriorated or affected in a dose-dependent manner with smoking, even after adjusting for covariates [5,7,79,80,81,82,83], while in other studies AHI seems not to be related to smoking [84,85,86,87,88,89,90,91]. As far as the Epworth sleepiness scale (ESS), the majority of the studies concluded that it is worse in smokers [79,81,84,86], although there were also a few notable exceptions in this topic [7,90].

Furthermore, it is rather clear that smokers with OSAHS present significantly more frequently cardiovascular, metabolic, respiratory and gastrointestinal co-morbidities compared to non-smokers [5,7,79,81,83,84,92]. Additionally, it seems that smoking ameliorates the beneficial effect of the treatment of OSAHS with continuous positive airway pressure (CPAP) [91]. Finally, it is worth mentioning that in a genetic study about smoking, coffee, alcohol and OSA, in the univariate mendelian regression, smoking initiation was associated with an increased risk of OSA incidence; however, in the multivariate model, this association was not significant after adjusting for BMI [93]. Table 2 shows the studies that investigated the relationship between smoking and OSAHS.

### 3.3. Smoking and Other Sleep Disorders

As shown previously, smoking is associated with increased sleep latency, difficulty in initiating and maintaining sleep, shorter total sleep time and earlier morning awakening, all of which are characteristic constituents of insomnia. Compared to nonsmoking, smoking was associated with experiencing increased insomnia, while night-time smoking was significantly associated with greater insomnia and shorter sleep duration [6]. In night shiftworkers, smoking was associated with insomnia after recent significant life events [66]. During the COVID-19 pandemic, the severity of insomnia index was associated with pain in the elderly female patients who smoked [94]. Insomnia was more frequent in patients with rheumatic conditions who were also smokers, with chronic pain being a suggestive intermediate liaison [95]. Smoking was associated with insomnia in a dose dependent manner in two more studies, either with [57], or without alcohol consumption [56], while cognitive-behavioral therapy for insomnia plus smoking cessation counseling improved insomnia symptoms in another study [96]. Nevertheless, in two other studies, light but not heavy smoking was associated with insomnia, after controlling for covariates [36,97]. Finally, in a recent genetic study, it was demonstrated that insomnia was positively correlated with both smoking initiation and the number of cigarettes per day [53].

A very rare type of parasomnia is that of sleep-related compulsive smoking behavior, which was described with sleep-related eating disorder [8,98]. Prenatal maternal smoking was independently associated with an increased risk of offspring adolescent parasomnias including walking and talking in sleep and nightmares [99]. In college students, sleep-related disorders have not been related to smoking in one study [100], however, in another study of patients attending a sleep clinic for suspected OSAHS, ever smokers, compared to never smokers, presented more frequent episodes of sleep talking, abnormal movements and restless sleep [7].

As far as periodic leg movements in sleep or restless legs syndrome, two studies have shown that these disorders deteriorate with smoking [26,101]; however, another study demonstrated that they are not related [10]. In the same study, bruxism was shown to worsen with smoking [10]. Bruxism also worsened with smoking in another study, in which it was accompanied by arousals, especially in the N1 sleep stage and the non-supine position, indicating increased sleep fragmentation [9]; however, it was not improved with smoking cessation in a different study [102].

The arousal index was significantly increased in current and former smokers compared to non-smokers [88]. Furthermore, the same applied in patients with sleep-related compulsive smoking behavior [8]. Moreover, relative arousals were also increased in smokers who abstained from smoking [103] and those who received varenicline for smoking cessation [104], although those who received 24 h nicotine patches experienced significantly less microarousals than those who received 16 h nicotine patches [105]. Finally, the arousal index was significantly decreased in maternal smoking infants, a factor that combined with the apneic episodes in this population might contribute to sudden infant death syndrome [106]. Table 3 shows the studies that investigated the relationship between smoking and other sleep disorders.

### 3.4. Smoking and Sleep Architecture

Sleep macro architecture refers to the basic structural organization of normal sleep. There are two types of sleep—rapid eye movement (REM) and non-REM sleep. Non-REM sleep is divided into three stages: the N1 stage (2–5% of total sleep time), the N2 stage (45–55% of total sleep time) and the N3 stage (10–20% of total sleep time), while REM sleep corresponds to 20–25% of total sleep time. Current smokers tend to present a faster sleep electroencephalogram activity with lower delta power, which has a dose-dependent negative association with smoking, in non-REM sleep compared with former and never smokers and higher alpha power compared with never smokers [3]. Evidence also exists about diminished sleep continuity and increased wake time after sleep onset [107]. As far as sleep stages, it seems that the N3 stage or slow-wave sleep is decreased in smokers, while the N1 and N2 stages are increased, changes that seem to be dose dependent, as they are more evident to heavy smokers compared to mild smokers, or to current smokers compared to former smokers [5,80,85,108]. In another study, smokers presented a shorter sleep period time, longer sleep latency and consequently a higher REM sleep density with no differences regarding parameters of spectral analysis of the sleep electroencephalogram as well as in the sleep efficiency measured by PSG [26]. During smoking abstinence, changes in sleep stages and awakenings have been observed [103]. Nicotine patches, especially the 24 h compared to the 16 h ones, significantly increase the proportion of slow-wave sleep, REM density and REM beta activities, and decrease REM latency and N2 sleep stage duration [105,109,110]. On the other hand, the administration of varenicline does not cause changes in sleep macro architecture (N1, N2, N3, REM, sleep efficiency, total sleep time) apart from prolongation of sleep latency, N2 and N3 latency [104]. Furthermore, uvulopalatopharyngoplasty improves the N1 and N3 sleep stages significantly in smokers with OSAHS [92]. Finally, infants of mothers who smoke, sleep less, present a higher proportion of active sleep and a lower proportion of quiet sleep and more wakefulness after sleep onset, while they display more body movements and more disturbed sleep [111]. Moreover, in such infants, gastroesophageal reflux emerges more frequently in the REM sleep stage [112]. Table 4 shows the studies that investigated the relationship between smoking and sleep architecture.

### 3.5. Smoking, Sleep and Other Medical Conditions

There are many medical conditions that are affected by the simultaneous existence of smoking with a sleep problem. Obesity is significantly correlated, positively with the number of cigarettes, and negatively with sleep duration [11,113]. Leptin levels tend to increase in active smokers with OSAHS after treatment with CPAP compared to non-smokers, probably because smoking acts as a predisposing factor to leptin resistance [91]. Moreover, orexin-A levels were significantly lower in never smokers with OSAHS compared to ex- or current smokers [114]. The co-existence of OSA or short sleep duration with smoking was related not only with excess body weight, but also with metabolic diseases such as resistance to insulin or type II diabetes mellitus, increased triglycerides, increased low-density lipoprotein (LDL) cholesterol and decreased high-density lipoprotein (HDL) cholesterol levels and also with cardiovascular diseases such as hypertension and coronary artery disease [9,12,13,81,83,115,116,117,118,119]. Furthermore, in patients with OSAHS, current smoking determines the circulating levels of myeloperoxidase (MPO), an oxidative stress marker, and matrix metalloproteinase-9 (MMP-9), a plaque destabilizer, both signaling a worse prognosis [120]. More particularly, MPO is a heme-group enzyme in azurophilic granules of neutrophiles and monocytes and works as a peroxidase, which triggers oxidative stress in inflammatory pathways, while MMP-9 is a zinc containing endopeptidase, essential in cardiac and vascular remodeling by degrading the extracellular matrix; increased circulating levels of both MPO and MMP-9 are associated with worse prognosis in patients with coronary artery disease [120]. In addition, OSA severity and smoking are independent predictors of peripheral arterial tonometry (PAT), a marker which quantifies endothelial dysfunction [121]. Nevertheless, a study on upper airway surgery in patients with OSAHS demonstrated that postoperative smoking does not worsen glycemic or lipid profile, which are improved with the surgery [92]. Finally, apart from coronary arteries, smoking and OSAHS seem to also insult the myocardium, since, in patients with systolic heart failure, they predispose to nocturnal ventricular arrythmias, that might be proved fatal [122].

Apart from metabolic and cardiovascular diseases, poor sleep combined with smoking participate in the pathophysiological mechanism of numerous medical conditions. They increase hemoglobulin (Hb) [5], and along with hyperlipidemia are risk factors for proteinuria among high altitude mountain trekkers [123], while they are related to lower levels of iron and magnesium [9]. Furthermore, they are related to increased cortisol levels, which in turn play a role in wake time after sleep onset [107], however, sleep, but not smoking, is related to serum testosterone levels and liver steatosis [124,125]. The central nervous system also seems to be affected by smoking and poor sleep. Smoking stimulates the release of dopamine and serotonin, which promote awakening and inhibit REM sleep, while dopamine and serotonin transporters play a key role in their reuptake from the presynaptic neurons. Dopamine was significantly increased, while dopamine transporter was significantly decreased in the cerebrospinal fluid (CSF) of active smokers with poor sleep [32]. Similarly, inflammatory markers such as tumor necrosis factor alpha (TNF-a) and interleukin-1b (IL-1b) were also increased [28]. TNF-a and IL-1b are proinflammatory cytokines, which play a central role in the amplification and orchestration of the inflammatory response. They modulate blood–brain barrier permeability and might be associated with poorer sleep quality in active smoking and central nervous system circadian dysregulation [28]. Perhaps these changes in CSF play a role in smokers with poor sleep quality and masticatory myofascial pain [126], pain related to rheumatic diseases [95], or COVID-19 [94]. In any case, the combination of smoking and poor sleep quality is not only a feature in the COVID-19 pandemic but also in HIV [46,127]. Furthermore, poor sleep quality along with several other unhealthy habits have been associated with lower grey matter brain volume and although smoking was not a significant factor in the multivariate analysis [128], it was associated with several neurological defects such as mild cognitive impairment, memory problems and even sudden deafness [15,27,129,130]. In addition, smoking combined with poor sleep quality has also been associated with mental health problems [54] such as anxiety, depression and bipolar disorder [22,45,50,131]. This has also been demonstrated in a genetic study, in which smoking and sleep quality were independent risk factors for both depression and bipolar disorder [16]. Perhaps, this is associated with the effect of nicotine in the cholinergic system [110]. In any case, poor sleep and smoking have also been related to other substance misuse, such as cannabis [56].

Another link between smoking and sleep with other medical conditions is that with respiratory system problems. In a study of children with asthma it was found that smoking of the caregivers leads to increased reliever medication use, which in turn disrupted the sleep quality of children [132]. However, another study demonstrated that OSAHS is a determinant of asthma control irrespective of smoking [133]. As far as chronic obstructive pulmonary disease (COPD), in patients with OSA and COPD who still smoke, smoking-related airway inflammation, is characterized by higher levels of exhaled CO and H_2_S and lower levels of NO, which consequently augments the effect of ozone on SpO_2_ during sleep [14]. Furthermore, patients with OSAHS who smoke have worse a forced expiratory volume in 1 s to forced vital capacity (FEV_1_/FVC) ratio and present more frequently with COPD [5,79], although this was not evident in another study [90].

Finally, a distinct mention is required for the relationship between smoking and sleep problems with gynecological and infant problems. In a genetic study about risk factors for breast cancer, sleep satisfaction was included among them, but smoking was not [134]. Sleep-disordered breathing also seems to be a risk factor for gestational diabetes mellitus, irrespective of smoking status [135]; however, smoking seems to induce several sleep problems in pregnant women such as difficulty in initiating or maintaining sleep, short sleep duration, insufficient sleep, poor sleep quality, early-morning awakening, excessive daytime sleepiness and tiredness and restless legs syndrome [17,101]. Even exposure to passive smoking seems to exacerbate such sleep disturbances [136], while postpartum behavioral interventions in sleep women helped to prevent them from relapsing in smoking [137]. Maternal smoking also has deleterious effects in the sleep of their infants. These infants sleep less overall, with a higher proportion of active sleep and a lower proportion of quiet sleep, and experience more wakefulness after sleep onset, with more body movements and, as a result, more disturbed sleep [18,111]. They also tend to have increased risk for GER during REM sleep [112], while they also are at greater risk for admitting to the neonatal intensive care unit [138]. Furthermore, when they grow up, they face an increased risk of adolescent parasomnias including sleep-walking, sleep-talking and nightmares, and an increased likelihood of reporting sleep problems at the 14 years of age [99]. Finally, maternal smoking along with supine sleep position are risk factors for sudden infant death syndrome [106,139,140,141,142]. Table 5 shows the studies that investigated the relationship between smoking, sleep and other medical conditions.

### 3.6. Smoking Cessation and Sleep

Sleep quality is an important factor in smoking cessation [143]. Sleep duration is positively associated with smoking cessation [144], while insomnia decreases the odds of successful smoking cessation [53]. Heavy smokers often suffer from nocturnal sleep-disturbing nicotine craving [2], while poor sleep quality during smoking cessation leads to increased withdrawal, craving, irritability, anxiety, tension and total smoking urges [4,103]. Sleep disorders are considered as withdrawal symptoms during cessation. Emotional disturbances such as anxiety and depression are common in those who experience poor sleep quality during smoking cessation and might be a target for cognitive behavioral treatment [69,131,145]. Olfactory aversive conditioning during sleep might reduce cigarette-smoking behavior in a sleep stage-dependent manner, persisting for several days [146]. Additionally, targeting sleep quality might improve smoking cessation odds [67,137]; however, this was not the case in two studies that try to implement this theory [96,147]. Smoking cessation also has not improved other sleep disorders, such as bruxism [102]. In a smoking cessation study, it was demonstrated that varenicline and transdermal nicotine patches might increase sleep disturbance, although they attenuate withdrawal symptoms unrelated to sleep compared to placebo [148]. Nevertheless, in another study, varenicline had little effect in sleep macro architecture in patients with OSA, although it did slightly increase the arousal index and decreased sleep efficiency [104]. Furthermore, the 24 h nicotine patch, compared to the 16 h one, improved sleep quality and decreased smoking urges [105,109,149]. To sum up, a quit smoking effort initially seems to lead to sleep disturbances; however, these disturbances could be addressed with targeted interventions, since smoking could be considered as the result of a delayed sleep phase, rather than a cause [150]. On the other hand, the long-term effects of smoking cessation on sleep quality seem to be particularly beneficial and lasting. Table 6 shows the studies that investigated the relationship between smoking cessation and sleep.

### 3.7. Limitations

One of the major limitations of this review is its reliance on cross-sectional studies, since longitudinal ones are necessary to establish casual relationships between smoking and sleep disturbances. Another major limitation is that the majority of the included studies of this review are potentially biased, such as self-reported smoking status and sleep quality, which could affect the reliability of the findings. Since there are several inconsistences in the literature regarding the participation of smoking in the pathogenesis of OSAHS, this can be considered as another limitation of the present review, which could be addressed by future cohort studies.

## 4. Conclusions

Smoking has a deleterious effect on sleep quality, while poor sleep quality also seems to increase the likelihood of smoking. It is not clear whether smoking participates in the pathogenesis of OSAHS; however, smokers with OSAHS present a more severe disease as far as their physiological measurements. Furthermore, smoking seems to exacerbate other sleep disorders such as insomnia, parasomnias, arousals, bruxism and restless legs, while it disrupts sleep architecture by promoting a less deep and consequently less restful and refreshing sleep. Alarmingly, poor sleep multiplies the effect of smoking in numerous medical conditions and vice versa, while good sleep might increase the likelihood of successful smoking cessation.

Since there is a lack of studies examining specific aspects of smoking, such as intensity, weekly regularity and diurnal timing, it would be highly beneficial to investigate, in future studies, how these factors affect circadian rhythm, providing valuable information for the relationship between smoking and sleep.

## Figures and Tables

**Table 1:** Studies that investigated the relationship between smoking and sleep quality.

Reference	Studies’ Main Findings
Soldatos CR et al. 1980 [19] Delasnerie-Laupretre N, et al. 1993 [20] Wetter DW et al. 1994 [21] Phillips BA et al. 1995 [22] Bale P et al. 1982 [23] Lexcen FJ et al. 1993 [24]	The last five decades, these studies had correlated smoking with various aspects of poor sleep quality, such as difficulty in initiating sleep, staying asleep and waking up, nightmares, snoring, excessive daytime sleepiness and minor accidents. Sleep duration was inversely correlated with smoking even in athletes, whilst the effects of smoking on sleep were partly attributed to the fact that smokers consume greater quantities of caffeine and alcohol as well.
Patten CA et al. 2000 [25]	Cigarette smoking status showed a dose–response relationship with development of sleep problems, frequent sleep problems and with persisting frequent sleep problems.
Jaehne A et al. 2012 [26] Liu JT et al. 2013 [27] Liu Y et al. 2020 [28] Woo DH et al. 2023 [1] Hwang JH et al. 2022 [30] Al-Mshari A et al. 2022 [31] Li H et al. 2020 [32]	Pittsburgh Sleep Quality Index (PSQI) scores were persistently higher in smokers compared to non-smokers, in both males and females, even after controlling for psychological factors, while a quantitative relation between sleep disturbance and smoking with significantly longer sleep latency in heavy smokers compared to non-smokers, also existed.
Peters EN et al. 2011 [29]	Individuals who both wake during the night to smoke and report clinically significant sleep disturbance represent a high-risk group of smokers.
Purani H et al. 2019 [4]	Poorer sleep quality was associated with increased withdrawal, craving and total smoking urges.
Bellatorre A et al. 2017 [33] Nuñez A et al. 2021 [6] Sahlin C et al. 2009 [34] McNamara JP et al. 2014 [35] Mak KK et al. 2010 [36] Mehari A et al. 2014 [37] Merianos AL et al. 2023 [38] Sujarwoto S et al. 2020 [39] AlRyalat SA et al. 2021 [40] Nasri O et al. 2021 [41] Banna MHA et al. 2023 [42] Nakata A et al. 2023 [43] Sabanayagam C et al. 2011 [44]	Apart from the PSQI, there are also other sleep parameters that present deterioration with smoking, such as difficulty in falling and staying asleep, longer sleep latency, shorter sleep time, often in a dose-dependent manner, or after second-hand smoke exposure.
Bilsky SA et al. 2016 [45] Bar-Zeev Y et al. 2023 [46] Merikanto I et al. 2017 [17] Patterson F et al. 2016 [47] Parkes KR. 2002 [48]	Other factors that seem to intervene in the relationship between smoking and sleep disturbances are stress, especially during stressful occasions like a pandemic, late chronotypes and night shiftwork.
Otsuka Y et al. 2022 [49] Hattatoğlu DG et al. 2021 [50]	These studies have not identified a relationship between smoking and sleep quality, apart from indirect confounding factors or effects in sleep hygiene.
Oh S et al. 2022 [51] Zhou B et al. 2018 [52] Gibson M et al. 2019 [53]	These studies have identified a significant relationship between smoking and sleep quality, by using biochemical or genetic examinations.
Metse AP et al. 2021 [54] Riera-Sampol A et al. 2022 [55] Leger D et al. 2022 [56] Hussain J et al. 2022 [57] Palmer CD et al. 1980 [58] Manzar MD et al. 2018 [59] Masood S et al. 2015 [60]	Another aspect in the relationship between smoking and poor sleep quality is their common effect on other unhealthy habits such as bad nutrition, caffeine intake, alcohol consumption, illicit drug usage and lack of physical activity.
Chang LY et al. 2018 [61] Sabatier T et al. 2023 [62] Oshri A et al. 2017 [63]	Studies that investigated the relationship between sleep problems in adolescence and subsequent smoking trajectories have resulted in conflicting evidence.
Patterson F et al. 2018 [64] Hamidovic A et al. 2009 [65] Kageyama T et al. 2005 [66] Nair US et al. 2019 [67] Chen H et al. 2017 [68] Fillo J et al. 2016 [69]	Sleep deprivation or a transition from adequate to inadequate sleep causes increased tobacco cigarette consumption, probably due to the expectance that nicotine might reduce subjective sleepiness, thus smoking cessation programs should target smokers with poor sleep and promote interventions in this direction.

**Table 2:** Studies that investigated the relationship between smoking and OSAHS.

Reference	Studies’ Main Findings
Kim KS et al. 2012 [70] Dülger S et al. 2021 [71] Virkkula P et al. 2005 [72]	Smoking might contribute to the pathogenesis of obstructive sleep apnea (OSA) through the increased thickness and edema of the uvular mucosa lamina propria and the increased total nasal resistance in a supine position in smokers.
Jang YS et al. 2023 [73] Zhang Q et al. 2007 [74] Wetter DW et al. 1994 [75] Kashyap R et al. 2001 [76]	Smokers present a higher odds ratio in a dose-dependent way for sleep-disordered breathing or OSA, adjusted for confounders such as age, sex, body mass index (BMI) and alcohol.
Ioannidou D et al. 2021 [13] Cohen O et al. 2019 [77] Esen AD et al. 2021 [78]	Smoking was not related to the prevalence of OSA, when adjusted for covariates, apart from in younger females or in patients with BMI < 30.
Yosunkaya S et al. 2021 [5] Grigoriou I et al. 2023 [7] Oțelea MR et al. 2022 [79] Varol Y et al. 2015 [80] Bielicki P et al. 2019 [81] Boussoffara L et al. 2013 [82] Porebska I et al. 2014 [83] Shao C et al. 2020 [84] Mauries S et al. 2023 [85] Wang X et al. 2021 [86] Casasola GG et al. 2002 [87] Conway SG et al. 2008 [88] Hoflstein V. 2002 [89] Ben Amar J et al. 2018 [90] Suzgun MA et al. 2023 [91]	Current smokers present OSA earlier and more severly with worse oxygen desaturation index (ODI), mean and minimum SaO_2_, total sleep time and sleep time ratio with SaO_2_ below 90%, mean apnea duration, nocturnal hypoxia index and COHb levels and the Epworth sleepiness scale (ESS), while the evidence for the relationship between smoking and apnea–hypopnea index (AHI) are conflicting with some studies showing a dose-dependent relationship, while others showing no relationship.
Zhu H et al. 2021 [92]	The postoperative improvement of sleep structure in non-smoking OSA patients was better than smokers.
Yang Y et al. 2023 [93]	Smoking initiation was associated with increased risk of OSA, while never smoking was associated with decreased risk of OSA.

**Table 3:** Studies that investigated the relationship between smoking and other sleep disorders.

Reference	Studies’ Main Findings
Nuñez A et al. 2021 [6] Kageyama T et al. 2005 [66] Eskici İlgin V et al. 2023 [94] Stipelman BA et al. 2013 [95] Hussain J et al. 2022 [57] Leger D et al. 2022 [56] Fucito LM et al. 2014 [96] Mak KK et al. 2010 [36] Riedel BW et al. 2004 [97] Gibson M et al. 2019 [53]	Compared to non-smoking, smoking was associated with experiencing increased insomnia, while night-time smoking was significantly associated with greater insomnia and shorter sleep duration with a dose-dependent manner in night shiftworkers, in patients with chronic pain and rheumatic diseases or during the pandemic, with or without alcohol consumption, after controlling for covariates or in genetic studies, while smoking cessation counseling improved insomnia symptoms.
Provini F et al. 2008 [8] Kazi SE et al. 2022 [98] O’Callaghan F et al. 2019 [99] Yahia N et al. 2017 [100] Grigoriou I et al. 2023 [7]	Smoking was related to various types of parasomnia such as compulsive eating disorder and compulsive smoking during sleep, sleepwalking, sleeptalking, nightmares, abnormal movements and restless sleep.
Jaehne A et al. 2012 [26] Kaneita Y et al. 2005 [101] Lavigne GL et al. 1997 [10] Frosztega W et al. 2022 [9] Ahlberg J et al. 2024 [102]	The evidence on the relationship between smoking and restless legs syndrome or bruxism is conflicting, with some studies showing worsening, and others showing no connection.
Conway SG et al. 2008 [88] Prosise GL et al. 1994 [103] Pataka A et al. 2021 [104] Staner L et al. 2006 [105] Sawnani H et al. 2004 [106]	The arousal index was significantly increased in current and former smokers and decreased in maternal smoking infants, while relative arousals were also increased in smokers who abstained from smoking or received treatment for smoking cessation.

**Table 4:** Studies that investigated the relationship between smoking and sleep architecture.

Reference	Studies’ Main Findings
Truong MK et al. 2021 [3]	Current smokers had lower delta power in non-rapid eye movement (REM) sleep and higher alpha power compared with never smokers.
Cohen A et al. 2019 [107]	Smoking is associated with reduced sleep continuity, something that may involve the hypothalamic–pituitary–adrenocortical axis.
Yosunkaya S et al. 2021 [5] Varol Y et al. 2015 [80] Mauries S et al. 2023 [85] Zhang L et al. 2006 [108]	The N3 stage or slow-wave sleep is decreased in smokers, while the N1 and N2 stages are increased, changes that seem to be dose dependent, as they are more evident in heavy smokers compared to mild smokers, or in current smokers compared to former smokers.
Jaehne A et al. 2012 [26]	Smokers had a shorter sleep period time and higher REM sleep density than non-smokers.
Prosise GL et al. 1994 [103]	The multiple sleep latency tests latency to stage 1 sleep decreased during smoking cessation.
Staner L et al. 2006 [105] Aubin HJ et al. 2006 [109] Salin-Pascual RJ. 2002 [110]	Nicotine patches, especially the 24 h compared to the 16 h ones, significantly increase the proportion of slow-wave sleep, REM density and REM beta activities, while decreasing REM latency and N2 sleep stage duration.
Pataka A et al. 2021 [104]	No significant differences were observed in sleep macro architecture treatment with Varenicline apart from prolongation of N2 and N3 latency in smokers.
Zhu H et al. 2021 [92]	Postoperative smoking was associated with worse sleep structure.
Stéphan-Blanchard E, et al. 2008 [111]	Neonates born to heavy-smoking mothers displayed disturbed sleep structure and continuity, higher proportion of active sleep and lower proportion of quiet sleep.
Djeddi D et al. 2018 [112]	Gastroesophageal reflux associated with smoking exposure was particularly obvious during REM sleep.

**Table 5:** Studies that investigated the relationship between smoking, sleep and other medical conditions.

Reference	Studies’ Main Findings
Alsulami S et al. 2023 [11] Aldahash FD et al. 2018 [113] Suzgun MA et al. 2023 [91] Aksu K et al. 2009 [114]	Obesity is positively correlated with the number of cigarettes and negatively correlated with sleep duration, smoking acts as a predisposing factor to leptin resistance in OSA patients, increasing its secretion, while orexin-A levels are significantly lower in smokers with OSA compared to ex- or current smokers.
Frosztega W et al. 2022 [9] Zhu H et al. 2017 [12] Ioannidou D et al. 2021 [13] Bielicki P et al. 2019 [81] Porebska I et al. 2014 [83] Li L et al. 2017 [115] Donovan LM et al. 2018 [116] Lavie L et al. 2008 [117] Oliveira G et al. 2019 [118] Blazejova K et al. 2000 [119] Zhu H et al. 2021 [92]	The co-existence of OSA or short sleep duration with smoking was related to metabolic diseases such as resistance to insulin or type II diabetes mellitus, increased triglycerides, increased low-density lipoprotein (LDL) cholesterol and decreased high-density lipoprotein (HDL) cholesterol levels and also with cardiovascular diseases such as hypertension and coronary artery disease.
Özkan E et al. 2023 [120] Lui MM et al. 2016 [121] Javaheri S et al. 2012 [122]	An oxidative stress marker, a plaque destabilizer and peripheral artery tonometry quantify endothelial dysfunction, while nocturnal vevtricular arrythmias might be proved fatal in smokers with OSA and coronary artery disease.
Yosunkaya S et al. 2021 [5] Wada K et al. 2006 [123] Cohen A et al. 2019 [107] Kirbas G et al. 2007 [124] Mikolasevic I et al. 2021 [125]	Poor sleep combined with smoking increase hemoglobulin (Hb) and along with hyperlipidemia are risk factors for proteinuria. They are related to lower levels of iron and magnesium and increased cortisol levels. However, sleep, but not smoking, is related to serum testosterone levels and liver steatosis.
Li H et al. 2020 [32] Liu Y et al. 2020 [28]	Poor sleep and smoking combined is associated with higher levels of dopamine and TNF-α in celebrospinal fluid.
Custodio L et al. 2015 [126] Stipelman BA et al. 2013 [95] Eskici İlgin V et al. 2023 [94] Bar-Zeev Y et al. 2023 [46] Patterson F et al. 2019 [127] Kokubun K et al. 2021 [128] Hu M et al. 2019 [15] Liu JT et al. 2013 [27] Lin YN et al. 2016 [129] Nakamura M et al. 2001 [130]	Smoking and poor sleep quality are related to masticatory myofascial pain, or pain related to rheumatic diseases, lower grey matter brain volume and several neurological defects, such as mild cognitive impairment, memory problems and even sudden deafness, or COVID-19 and HIV complications.
Metse AP et al. 2013 [54] Phillips BA et al. 1995 [22] Bilsky SA et al. 2016 [45] Hattatoğlu DG et al. 2021 [50] Hahad O et al. 2022 [131] He M et al. 2023 [16] Salin-Pascual RJ. 2002 [110] Leger D et al. 2022 [56]	Smoking combined with poor sleep quality have been associated with mental health problems such as anxiety, depression and bipolar disorder, while they have also been related to substance misuse, such as cannabis.
Miadich SA et al. 2018 [132] Özden Mat D et al. 2021 [133] Zhang W et al. 2023 [14] Oțelea MR et al. 2022 [79] Ben Amar J et al. 2018 [90]	Smoking and poor sleep quality are related to asthma control, while in chronic obstructive pulmonary disease (COPD), smoking-related airway inflammation is characterized by higher levels of exhaled CO and H_2_S and lower levels of NO, which consequently augments the effect of ozone on SpO_2_ during sleep. Also, patients with OSA who smoke have worse respiratory function and present more frequently with COPD.
Yu LX et al. 2021 [134] Teni MT et al. 2022 [135] Merikanto I et al. 2017 [17] Kaneita Y et al. 2005 [101] Ohida T et al. 2007 [136] Stone KC. 2023 [137]	Smoking and sleep quality combined are not related to breast cancer; however, they are related, even with passive smoking, to several problems in pregnant women such as gestational diabetes, difficulty in initiating or maintaining sleep, short sleep duration, insufficient sleep, poor sleep quality early-morning awakening, excessive daytime sleepiness and restless legs syndrome, while postpartum interventions in sleep prevent smoking relapse.
Mennella JA et al. 2007 [18] Stéphan-Blanchard E et al. 2008 [111] Djeddi D et al. 2018 [112] Hannan KE et al. 2020 [138] O’Callaghan F et al. 2019 [99] Sawnani H et al. 2004 [106] Horne RS et al. 2002 [139] Nelson EA et al. 2001 [140] Anderson ME et al. 2005 [141] Tirosh E et al. 1996 [142]	Infants of smoking mothers sleep less, with lower proportion of quiet sleep, more wakefulness after sleep onset, more body movements and more disturbed sleep. They also have increased risk of gastroesophageal reflux (GER) during REM sleep, neonatal intensive care unit admission, adolescent parasomnias, or other sleep problems in general, while maternal smoking along with supine sleep position are risk factors for sudden infant death syndrome.

**Table 6:** Studies that investigated the relationship between smoking cessation and sleep.

Reference	Studies’ Main Findings
Peltier MR et al. 2017 [143] Rapp K et al. 2007 [144] Gibson M et al. 2019 [53]	Sleep duration is positively associated with smoking cessation, while insomnia decreases the odds of successful smoking cessation.
Riemerth A et al. 2009 [2] Purani H et al. 2019 [4] Prosise GL et al. 1994 [103] Fillo J et al. 2016 [69] Hahad O et al. 2022 [131] Farris SG et al. 2020 [145] Arzi A et al. 2014 [146] Nair US et al. 2019 [67] Stone KC. 2023 [137] Fucito LM et al. 2014 [96] Okun ML et al. 2011 [147] Ashare RL et al. 2017 [148]	Heavy smokers often suffer from nocturnal nicotine craving, while poor sleep quality during smoking cessation efforts leads to increased withdrawal, craving, irritability, anxiety, tension and smoking urges. Sleep disorders are considered withdrawal symptoms during smoking cessation, while emotional disturbances such as anxiety and depression are common in those who experience poor sleep quality during smoking cessation and might be a target for cognitive behavioral treatment.
Ahlberg J et al. 2024 [102]	Smoking cessation is not associated with a decline in reported sleep bruxism.
Pataka A et al. 2021 [104]	Varenicline treatment worsened sleep quality as a prolongation of sleep latency, N2 and N3 latency was observed.
Staner L et al. 2006 [105] Aubin HJ et al. 2006 [109] Wolter TD et al. 1996 [149]	The 24 h nicotine patch, compared to the 16 h one, improved sleep quality and decreased smoking urges.
Ghotbi N et al. 2023 [150]	Smoking may be a consequence of, rather than a cause, for social jetlag, while daytime sleepiness is a significant predictor of outcome, but did not improve with cessation.
